# Genetic Diversity and Genetic Structure of Different Types of Natural Populations in *Osmanthus fragrans* Lour. and the Relationships with Sex Ratio, Population Structure, and Geographic Isolation

**DOI:** 10.1155/2014/817080

**Published:** 2014-11-10

**Authors:** Shaoqing Hu, Shuai Wu, Yiguang Wang, Hongbo Zhao, Yuanyan Zhang

**Affiliations:** ^1^College of Civil Engineering and Architecture, Zhejiang Sci-Tech University, Hangzhou 310018, China; ^2^Department of Ornamental Horticulture, School of Landscape Architecture, Zhejiang Agriculture and Forestry University, Lin'an, Hangzhou 311300, China

## Abstract

*Osmanthus fragrans* Lour., an evergreen small tree, has the rare sexual system of androdioecy (coexistence of males and hermaphrodites), once with wide-spread natural distribution in the areas of the South Yangzi river basin. However, due to excessive human utilization, natural distribution became fragmented and the number and size of natural populations reduced sharply. With four different types of natural populations from the same region as research object, we aim to provide a comparative analysis on the relationships among genetic diversity, sexual system, population structure and size, and geographic isolation by ISSR. In genetic parameters of *N*_*e*_, *H*_*e*_, and *I*, the LQGC population had the highest value and the LQZGQ population had the lowest value. These indicated that LQGC population showed the highest genetic diversity, followed by QDH and JN population, and LQZGQ population exhibited the lowest genetic diversity. Genetic diversity in populations is closely related to population structure, reproduction mode, and sex ratio. However, there seems to be no obvious correlation between genetic diversity and population size. The results of AMOVA showed that genetic variations mostly occurred within populations. It indicates that no significant genetic differentiation among populations occurs, and geographic isolation has no significant effect on genetic diversity.

## 1. Introduction 

*Osmanthus fragrans* Lour. (Oleaceae), an evergreen small tree with the sexual system of androdioecy (coexistence of males and hermaphrodites in natural populations), is one of the most important ornamental plants and is also a famous aromatic plant in China. The species has been utilized for many years and has a long history of cultivation (more than 2500 years) [1].* Osmanthus fragrans* consists of four cultivar groups, namely, Aurantiacus, Albus, Lutes, and Asiaticus, with approximately 120 cultivars [1, 2]. The cultivars are mostly produced from artificial selection from natural populations and cultivar groups. Wild germplasm, which exhibits high morphological and genetic variation, is an important gene bank that could be useful for breeding improvement [3]. However, excessive exploitation and utilization for natural resources have resulted in severe decline in the number and size of natural populations and damage and loss of the suitable habitats. The distribution range of the natural populations has been sharply reduced. Comprehensive analysis on genetic diversity of natural populations is necessary for the further utilization and protection of wild resources in this species.

China is a center of distribution and origin of* O. fragrans*. According to Chang et al. [4],* O. fragrans* is mainly distributed in the southwest area of China. However, with gradual in-depth field investigation, natural populations were found to be distributed in the south area of Yangzi River basin, especially in Zhejiang, Fujian, Hunan, Jiangxi, Guizhou, Guangxi, and Guangdong provinces [5–10]. However, because of severe damage to natural populations, current populations exist only in fragmented distribution and are left alive only in some resort areas, natural reserves, and inaccessible remote areas. Obvious geographic isolation also occurs among populations.

The genetic diversity in plants is not only related to internal genetic background and reproductive system but also affected by population structure, spatial distribution pattern, and reproduction mode. In* O. fragrans*, sex ratios of natural populations are mostly 1 : 1 (male : hermaphrodite) [11]. The genetic effects of habitat fragmentation on plant populations include the sampling effect in habitat fragmentation and the subsequent small-population effects [12–14]. Habitat fragmentation reduces genetic variation within populations and increases the genetic differentiation among populations. Meanwhile, genetic effects of habitat fragmentation on plant populations are influenced by generation length, fragmentation time, and population size. Investigation to remaining populations revealed that* O. fragrans* is distributed in evergreen broad-leaf forests in limestone mountain areas [5–10]. Natural reproduction modes include sexual reproduction and clonal propagation. In general, populations of clonal plants exhibit considerable levels of genetic diversity [15–18].

Intersimple sequence repeat (ISSR) has been widely used in researches on genetic diversity of natural populations of woody plants [19–21]. Relative researches using ISSR to analyze genetic relationship of cultivars in* O. fragrans* have been reported [22–24]. This study utilizes ISSR method to analyze the genetic diversity and genetic structure of four different types of natural populations from the same region in Zhejiang Province. We aim to analyze the relationships among genetic diversity, sexual system, population structure, and geographic isolation in* O. fragrans* and provide the theoretical basis for the protection of natural populations.

## 2. Materials and Methods

### 2.1. Sample Collection

**Figure 1:**
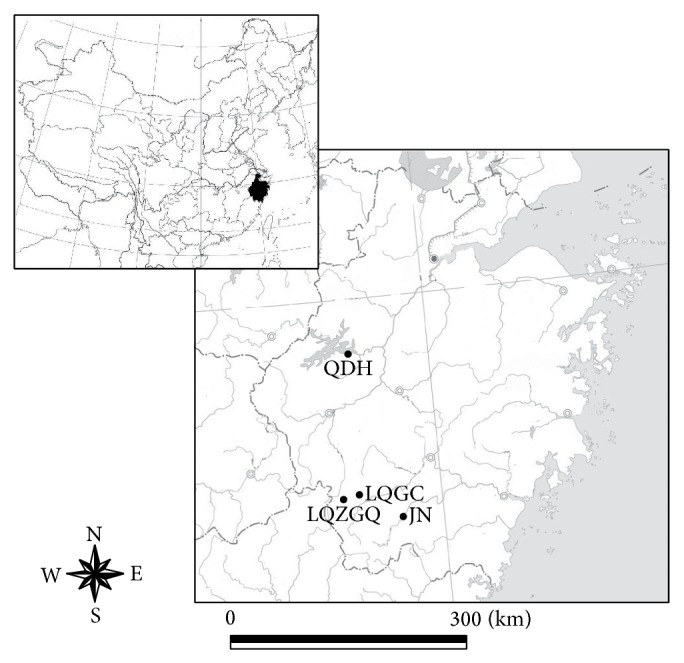
Study sites and distribution of* O. fragrans*.

A total of 188 samples from four natural populations in Zhejiang Province were collected from Jinning (JN), Longquan (Jinxi, LQZGQ; Daotai, LDGC), and Jiande (Thousand-Island Lake, QDH) (Table 1 and Figure 1). Approximately 10 g of fresh leaves was collected and placed quickly in the Ziploc bags with allochroic silicagel. The proportion of silicagel to leaves was at least 10 : 1 (w/w). Samples were brought to the laboratory and stored at −20°C after complete desiccation.

### 2.2. Population Type

The QDH population is basically pure stand of* O. fragrans*, with a large distribution density. Individuals were distributed closely and continuously, with most intervals being less than 0.5 m. Since regeneration seedlings were observed in the forest, the primary reproduction style focused on sexual reproduction. The proportion of male and hermaphroditic individuals meets the ratio of 1 : 1. The LGQC population was distributed in evergreen broad-leaves forest in active limestone mountains and individuals of* O. fragrans* in a patchy shape were distributed around the forest gaps. The intervals between individuals were more than 1.5 m. A certain quantity of clonal seedlings was found in the forest. Population reproduction included both clonal propagation and seed reproduction. The proportion of male to hermaphroditic individuals was 1 : 1. The LQZGQ population was distributed in evergreen broad-leaves forest also in active scree mountains. The individuals were all hermaphrodite, and the intervals between two individuals were 1 to 5 m. The JN population also with 1 : 1 sex ratio was distributed in evergreen broad-leaves forest with a fragmented distribution, in which severe man-made damage had occurred.

#### 2.2.1. DNA Extraction and PCR Amplification

Modified CTAB method [25] was used to extract total DNA from dried leaves. Primers were designed according to the sequences issued by British Columbia University of Canada. Referring to previous studies [22, 23], primers were synthesized by Shanghai Sangon Biotech Co., Ltd. A total of 15 primers with high polymorphism and good repeatability from the synthesized primers were screened for subsequent amplification. Reagents included the following: 10x loading buffer (containing Mg^2+^), Taq DNA polymerase, dNTPs, and 2000 bp DNA marker for amplification (TAKARA Biotech Co., Ltd.). The following amplification profile was used: predenaturation at 94°C for 5 min; denaturation at 94°C for 1 min, annealing at 50°C to 57°C for 45 s, and extension at 72°C for 90 s, with 32 cycles; extension at 72°C for 8 min and holding at 4°C. The PCR amplification reaction was performed on a PTC-100TM PCR instrument (German Biometra Company). The amplified product was subjected to electrophoresis on 1.5% agarose gel. After electrophoresis, the gel was photographed using gel imaging analyzer (US Bio-rad Company).

#### 2.2.2. Data Statistics Analysis

According to the bands in the electrophoretogram, the positions with the same migration rate on the gel and with DNA bands were recorded as “1,” and those without DNA bands were recorded as “0.” POPGENE 1.32 software [26] was used to calculate the genetic parameters: (1) percentage of polymorphic loci (PPL); (2) number of alleles (*N*_*a*_) and number of effective alleles (*N*_*e*_); (3) expected heterozygosity (*H*_*e*_); (4) Shannon's Information Index (*I*). GenAlEx 6.41 [27] software was used to determine molecular variance (AMOVA) and perform principal component analysis (PCA). AMOVA was used to calculate genetic variation. PCA was further carried out to verify and analyze the natural genetic clusters among populations and individuals. Genetic differentiation index of PhiPT (*Ф*_*st*_) among populations, Nei's genetic distance (*D*), and genetic identity (*I*_*N*_) were also calculated. The number of population migrants per generation, which reflects the gene flow level, was also calculated based on the following formula: *N*_*m*_ = (1 − *F*_*st*_)/4*F*_*st*_ [28].

## 3. Results

### 3.1. Band Polymorphisms

**Figure 2:**
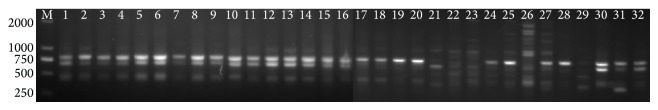
The amplification diagram of primer 198 in LQZGQ (1–16) and LQGC (17–32) populations. The band patterns among different individuals (1–16) in LQZGQ population were similar but those among different individuals (17–32) in LQGC population were polymorphic.

An average of 4.27 bands with molecular weights ranging from 200 bp to 2000 bp was amplified for each primer. The number of total bands from QDH population was the highest (63), and that from LQZGQ population was the lowest (39) and did not show any specific bands. The proportions of polymorphic loci (PPL) in populations were different, among which QDH population was the highest (96.92%) and LQZGQ population was the lowest (55.38%) (Table 2). The PPL among populations were ranked in the following descending order of QDH > LQGC > JN > LQZGQ. In LQZGQ population, some individuals had the same band patterns (Figure 2). It further verified the existence of clonal propagation. The five specific bands in QDH population were the highest and a specific band was found in the JN population, while LQGC and LQZGQ population presented no special band (Table 2).

### 3.2. Genetic Diversity and Genetic Structure

Shannon's Information Index (*I*) as well as the expected heterozygosity (*H*_*e*_) of all populations was ranked in the following descending order of LQGC > QDH > JN > LQZGQ, with an average of 0.332 and 0.217, respectively (Table 2). The estimated allele frequency with number of different alleles (*N*_*a*_) of QDH population was the highest (1.938) and that of LQZGQ population was the lowest (1.154), and the estimated allele frequency with number of effective alleles (*N*_*e*_) of LQGC population was the highest (1.406) and that of LQZGQ population was the lowest (1.331). According to the results of molecular variance analysis (AMOVA), genetic variance mostly occurred within populations and accounted for 85% of the total genetic variance (Table 3). The results indicate that the genetic variance was mainly attributed to genetic diversity within populations.

### 3.3. Genetic Differentiation

The total genetic differentiation coefficient of the four populations was 0.148 (Table 3), which implies that the genetic differentiation among populations is small. Among populations, genetic differentiation coefficients of JN and LQZGQ population were the highest (0.339) and exhibited the lowest gene flow (0.487) (Table 4). The genetic differentiation coefficients between LQGC and QDH population were the lowest (0.091) and exhibited the highest gene flow (2.510). Nei's genetic distance and genetic identity also showed the same trends (Table 5).

### 3.4. Principal Component Analysis

**Figure 3:**
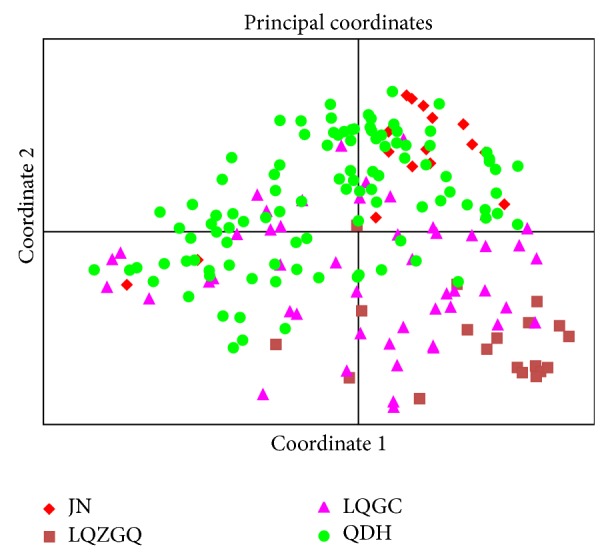
Principal coordinate analysis (PCA) of genetic differences among individuals of four natural populations in* O. fragrans*.

Individuals belonging to LQGC population exhibited a scattered distribution (Figure 3). The results indicate that the degree of genetic variation within population was the highest and contained the richest genetic information. The QDH population had the most number of individuals with scattered distribution. Results indicate that the degree of genetic differentiation within the QDH population was high and carried rich genetic information. The JN population was overlapped by QDH population, suggesting a close genetic relationship between these two populations. Most individuals belonging to LQZGQ population gathered independently. Compared with other populations, special genetic information existed in this population.

## 4. Discussion

### 4.1. Genetic Diversity in Species Level

The population genetic structure in plants depends not only on its genetic background and mating system but also on genetic drift, gene flow, natural selection, and so forth [29–31]. The amplification results of 15 ISSR primers for 188 samples of four natural populations indicated high genetic diversity at the species level, and genetic variance mainly occurs within the population. The total *H*_*e*_ of four populations was 0.217, which is consistent with the average genetic diversity index of many plants based on the ISSR molecular marker and is also consistent with the average genetic diversity index of widespread plants based on RAPD but is lower than that for long-lived perennial plants based on RAPD [32]. The total genetic differentiation coefficient among populations was 0.148, which is lower than the average value of 12 species based on the RAPD marker (*G*_*st*_ = 0.21) [33] and also lower than the average of the nine widely distributed species (*G*_*st*_ = 0.33) [32]. The results indicate that genetic differentiation among populations was not significant and strong gene flow existed among populations, which could ensure genetic information exchange among populations to maintain high genetic diversity at the species level. The results also indicate that habitat fragmentation did not significantly affect genetic structure.

### 4.2. Genetic Diversity among Populations

A certain degree of genetic difference existed among populations. In terms of PPL, the QDH population was the highest, and LQZGQ was the lowest. This value is lower than PPL of 19 cultivars by ISSR [23], but higher than that of 23 cultivars based on RAPD [34, 35] and that of 22 cultivars based on AFLP [36]. The *H*_*e*_ of the four populations ranged from 0.189 to 0.241, and the *I* ranged from 0.282 to 0.366. Among four populations, LQGC showed the highest genetic diversity, followed by the QDH and JN populations. The LQZGQ population exhibited the lowest genetic diversity.

**Figure 4:**
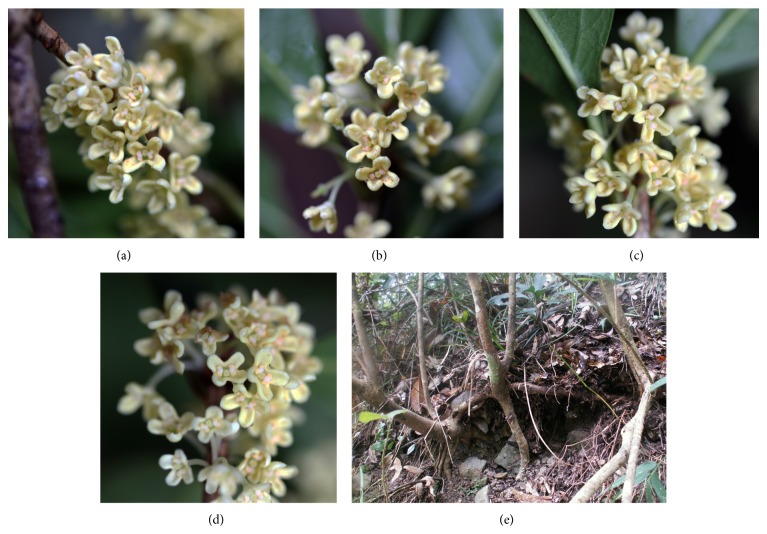
Floral traits of four individuals (a–d) and clone reproduction (e) in LQZGQ population.

**Figure 5:**
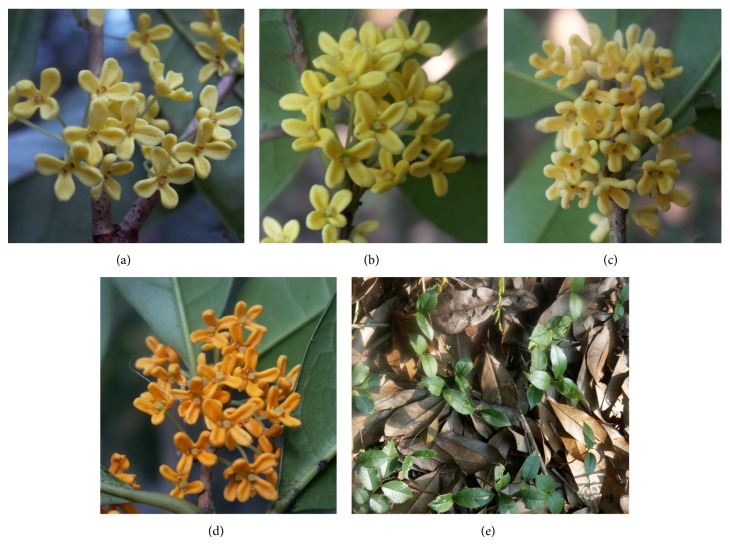
Floral traits of four individuals (a–d) and sexual reproduction (e) in QDH population.

Field investigations showed significant differences in population structure (distribution range, density, and age composition), regeneration mode, and sex ratio among these four populations. Sex ratios (males : hermaphrodites) in LQGC, QDH, and JN population were all 1 : 1 [11]. All individuals in LQZGQ population were hermaphrodite, with similar floral traits (Figures 4(a)–4(d)). The population site is an easily slipping scree slope. In the forest, a lot of clonal seedlings around adult trees were found (Figure 4(e)). Therefore, clonal propagation could be the main reproduction mode in LQZGQ population, which was further confirmed by the band patterns of this population. Genetic diversity of this population was considerably lower than the other three populations. In LQGC population, comparing with the other populations, most of the individuals have relatively older tree-age (the thicker trunk indicated the older age) and farther distribution interval between each other. Meanwhile, rich variations of floral traits existed among individuals. Clonal propagation was also one of reproduction modes in this population cooccurring with seed reproduction. The stable population structure maintained high genetic diversity in this population. In the clonal plant* Geum reptans*, clonal reproduction did not cause severe consequences for population genetic variability and neither did older age or higher elevation of the populations; gene flow and repeated seedling recruitment during succession could have been more frequent than commonly suggested [18]. The QDH population had largest distribution area and population size and exhibited the greatest distribution density with small intervals. Plentiful sexual seedlings were found within the population (Figure 5(e)). It indicated that the main regeneration mode of this population should be sexual reproduction. Rich floral variations among individuals also existed within the population (Figures 5(a)–5(d)). However, due to the lack of older-age trees, relatively simple population structure comparing to LQGC population led to lower genetic diversity. The JN population with wide but fragmented distribution exhibited similar and overlapped genetic diversity with QDH population (Table 2 and Figure 3). Although the size of JN population was very small due to serious destruction, it still exhibited relatively high genetic diversity. It indicated that this population still bore genetic information passed on from the original populations. In conclusion, sex ratio, regeneration mode, and population structure especially age composition played great roles in the maintenance of genetic diversity of natural populations in* O. fragrans*; however, population size and geographic isolation due to habitat fragmentation seemed to have no obvious influence on genetic diversity.

### 4.3. Protection for Natural Populations

In* O. fragrans*, genetic variances of natural populations mostly existed within populations and genetic differentiation among populations was small. Among populations, LQGC showed the highest genetic diversity and LQZGQ population exhibited the lowest genetic diversity. Therefore, as a protective strategy, high genetic diversity populations and populations carrying special genetic information should be protected primarily. The LQGC population had the highest genetic diversity, complicated population structure, and rich individual trait variations. The QDH population was distributed in limestone areas with relatively stable habitats, whose genetic diversity was also high and contained the most specific bands. The PCA results indicated that LQGC and QDH populations contained almost all genetic information of all populations carried. Consequently, the LQGC and QDH populations should be protected primarily. Simultaneously, the PCA results and amplified specific bands showed that LQZGQ carried the specific genetic information being absent in the other three populations. Thus, protecting LQZGQ population will help in the preservation and analysis of genetic diversity in this species.

## Figures and Tables

**Table 1:** Locations and distributions of four natural populations in *O. fragrans*.

Population	Location	Coordinate	Site	Area/hm^2^	Number of samples
QDH	Thousand-Island Lake, Jiande	29.531°N, 119.139°E	Limestone mountain	16.00	103
LQGC	Daotai, Longquan	28.183°N, 119.250°E	Limestone mountain	1.20	50
LQZGQ	Jingxi, Longquan	28.100°N, 118.883°E	Scree mountain	0.13	19
JN	Dajun, Jingning	28.183°N, 119.183°E	Limestone mountain	8.50	16

**Table 2:** The number of total bands (NTB) and private bands (NPB) in respective population, percentage of polymorphic loci (PPL), estimated allele frequency with number of different alleles (*N*_*a*_), number of effective alleles (*N*_*e*_), Shannon's Information Index (*I*), and expected heterozygosity (*H*_*e*_) in *O. fragrans*.

Population	NTB	NPB	PPL (%)	*N*_*a*_	*N*_*e*_	*I*	*H*_*e*_
JN	48	1	72.31	1.462 ± 0.110	1.371 ± 0.047	0.333 ± 0.033	0.218 ± 0.024
LQZGQ	39	0	55.38	1.154 ± 0.121	1.331 ± 0.049	0.282 ± 0.036	0.189 ± 0.026
LQGC	54	0	83.08	1.662 ± 0.094	1.406 ± 0.045	0.366 ± 0.033	0.241 ± 0.024
QDH	63	5	96.92	1.938 ± 0.043	1.358 ± 0.043	0.346 ± 0.030	0.219 ± 0.022

Total	65	—	100.0	1.554 ± 0.051	1.366 ± 0.023	0.332 ± 0.017	0.217 ± 0.012

**Table 3:** The analysis of molecular variance (AMOVA) of natural populations in *O. fragrans*.

Source	df	Sums of squares	MS	Variance component	Variation (%)	PhiPT	*P*
Among populations	3	190.773	63.591	1.443	15	0.148	<0.001
Within populations	184	1523.717	8.281	8.281	85		<0.001

Total	187	1714.489		9.724	100		

**Table 4:** Genetic differentiation of PhiPT analysis (below the diagonal) and gene flow (*N*_*m*_) (above the diagonal) among different populations in *O. fragrans*.

Population	JN	LQZGQ	LQGC	QDH
JN	—	0.487	1.295	2.028
LQZGQ	0.339	—	1.141	0.723
LQGC	0.162	0.180	—	2.510
QDH	0.110	0.257	0.091	—

**Table 5:** Nei's genetic distance (below the diagonal) and Nei's genetic identity (above the diagonal) of natural populations in *O. fragrans*.

Population	JN	LQZGQ	LQGC	QDH
JN	—	0.908	0.952	0.969
LQZGQ	0.096	—	0.935	0.921
LQGC	0.050	0.067	—	0.979
QDH	0.032	0.083	0.021	—
